# Acute Physiological and Psychological Effects of Qigong Exercise in Older Practitioners

**DOI:** 10.1155/2018/4960978

**Published:** 2018-04-02

**Authors:** Chun-Yi Lin, Tze-Taur Wei, Chen-Chen Wang, Wan-Chen Chen, Yu-Min Wang, Song-Yen Tsai

**Affiliations:** ^1^School of Medicine, College of Medicine, Fu Jen Catholic University, No. 510, Zhongzheng Rd., Xinzhuang District, New Taipei City, Taiwan; ^2^Research Assistant Center, Show Chwan Memorial Hospital, No. 542, Sec. 1, Chung-Shan Rd., Changhua, Taiwan; ^3^Department of Nuclear Medicine, Show Chwan Memorial Hospital, No. 542, Sec. 1, Chung-Shan Rd., Changhua, Taiwan; ^4^Society of Chinese Bioenergy Medical Qigong, No. 130-1, Nanyuan St., North District, Tainan City, Taiwan; ^5^Naturopathic Medicine Research Center, Changhua Christian Hospital, No. 135 Nanhsiao Street, Changhua City, Taiwan

## Abstract

Qigong is a gentle exercise that promotes health and well-being. This study evaluated the acute physiological and psychological effects of one session of qigong exercise in older practitioners. A total of 45 participants (mean age, 65.14 years) were recruited. Meridian electrical conductance, State-Trait Anxiety Inventory (STAI), heart rate variability (HRV), and Short Form 36 (SF-36) were evaluated and compared before and after one session of qigong exercise. The results revealed that the electrical conductance of all meridians, except spleen and bladder meridians, increased significantly (*p* < 0.05). Compared with baseline values, upper to lower body ratio and sympathetic/vagal index were significantly improved and closer to 1 (*p* = 0.011 and *p* = 0.007, resp.). STAI-S and STAI-T scores decreased significantly (*p* < 0.001 and *p* = 0.001, resp.). The RR interval of HRV decreased significantly (*p* = 0.035), a significant positive correlation was observed between kidney meridian electrical conductance and SF-36 physical scores (*r* = 0.74, *p* = 0.018), and a positive correlation was observed between pericardium meridian electrical conductance and SF-36 mental scores (*r* = 0.50, *p* = 0.06). In conclusion, one session of qigong exercise increased meridian electrical conductance, reduced anxiety, and improved body and autonomic nervous system balance. These findings provide scientific evidence for acute physiological and psychological effects of qigong exercise in older practitioners.

## 1. Introduction

Qigong, an ancient Chinese healing practice that was originally a martial art, has been developed and used to improve physical fitness and strength in China for 7000 years [1–4]. The word “qigong” involves two theories: “qi,” the vital energy of the body and “gong,” the training or cultivation of qi [5]. Concentration, relaxation, meditation, breathing regulation, and body posture and movements are the basic components of qigong [1, 2]. In contrast to conventional exercise, qigong aims to achieve a harmonious flow of qi in the body and cultivate a spirit of systematic training exercise to improve physical fitness and overall well-being through the coordination of rhythmic movements, regulated breathing, and meditation [6]. There are essentially two types of qigong: internal and external. Internal qigong or qigong exercise is self-directed and involves the use of movements, meditation, and controlled breathing patterns, whereas external qigong is typically performed by experienced practitioners who use their hands to direct qi onto patients for healing or treatment [1, 7–9]. Typically, qigong can be classified into dynamic and static qigong. Dynamic qigong involves the coordination of movements and meditation, whereas static qigong focuses on mental concentration and body relaxation without physical movements [1, 2].

Meridians are associated with the underdifferentiated, interconnected cellular networks that regulate growth and physiology. The meridian theory explains the distribution and nonspecific activation of organizing centers, acupuncture points, and the high electrical conductance of the meridians [10–13]. In the human body, meridian theory deals with physical regulation and pathological changes [14, 15]. The condition of the meridian system can be measured by its electrical characteristics which have been studied since the 1950s. Acupuncture points have been widely accepted as areas of low electrical impedance and high conductance [16–20]. Skin conductance response is typically measured by applying a small direct current signal through two electrodes placed on the skin [13, 21]. Nakatani identified 12 channels of high electrical conductance on the left and right sides of the body (24 in total) and developed the Ryodoraku theory according to the philosophy of traditional Chinese medicine (TCM) [16]. Skin electrical conductance varies with the activity of a subject's autonomic nervous system and has been used to investigate mood changes and the mechanisms of the autonomic nervous system [22, 23]. The properties of meridians reflect the conditions of certain organs when their mutual relations and changes with microelectrical current are analyzed and compared [24]. The electrical information mapping transforms associated with various medical syndromes, signs, symptoms, and diseases were reported in a previous study [13].

**Figure 1:**
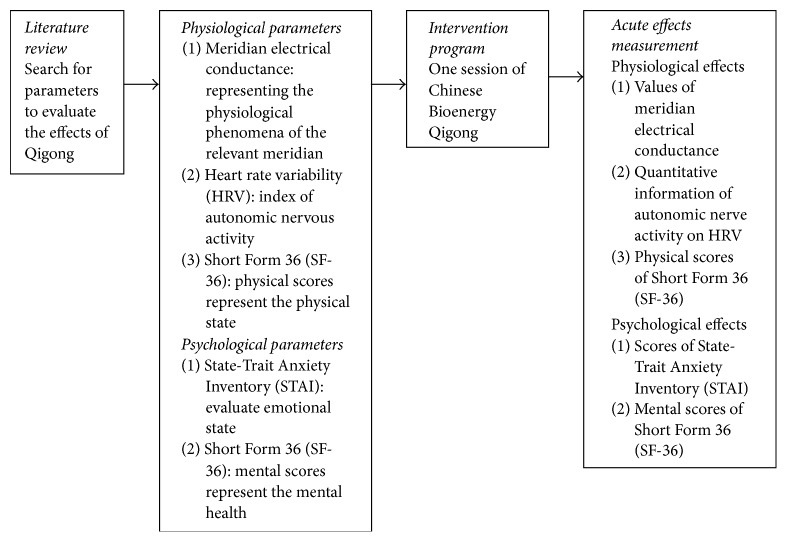
Conceptual framework.

The meridian electrical conductance investigates the meridian energy. Meridian energy analysis devices can reflex the conditions of certain organs through analysis of the symmetrical Yuan points. Changes with microelectrical currents represent the physiological phenomena of the relevant meridian [24, 25]. The electrical conductivity has been reported to monitor the effects of qigong workshop in the previous study [5]. Heart rate variability (HRV) is used as a sensitive index of autonomic nerve activity. The analysis of HRV provides quantitative information on autonomic control mechanisms [26]. It has been reported that five minutes of Tai Chi Qigong was found to improve HRV in nasopharyngeal cancer patients [27]. The State-Trait Anxiety Inventory (STAI) is a psychological inventory based on a 4-point Likert scale. It consists of forty questions on a self-report basis. These questionnaires are a highly reliable measure of stress and anxiety [28]. Qigong exercise significantly relieved anxiety and reduced stress among healthy people were reported in the previous study [29]. The SF-36 health survey is a self-report survey commonly used as a generic health questionnaire for adults which covers eight health domains, namely, physical functioning, physical role functioning, bodily pain, general health perceptions, vitality, social role functioning, emotional role functioning, and mental health [30]. SF-36 scores have been used to evaluate the effects of qigong exercise in breast cancer survivors [31]. The conceptual framework of this study was shown in Figure 1 guiding this study.

Long-term practice of qigong exercise may help in maintaining satisfactory health and preventing and treating illness. Qigong exercise has been reported to influence depression, stress, anxiety symptoms, chronic pain, immunity, infection, and quality of life [9, 32–36]. However, evidence-based research on the acute physiological and psychological effects of qigong exercise in older practitioners is yet to be reported. This study therefore evaluated the acute physiological and psychological effects of one session of qigong exercise in older practitioners.

## 2. Methods and Measures

### 2.1. Study Design

This research involved a prospective, pre- and postcomparison study. After obtaining informed consent from the participants, meridian electrical conductance measurements, questionnaires, and baseline heart rate variability (HRV) measurements were obtained before and after one session of qigong exercise.

Chinese Bioenergy Qigong is a popular and well known exercise in Taiwan and Hong Kong. It is easy to learn with low cost. There is no limitation in time or place to practice. Hundred thousand people practice Chinese Bioenergy Qigong in Taiwan. It not only active body adjustment but also harmonizes mind adjustment. It is easy for general population to practice every day. Chinese Bioenergy Qigong is a 1-hour session of qigong exercise conducted and popularized by Dr. Li Cheng Chung, which includes warm-up movements, breathing regulation, gentle movements, body stretching, meditation, and relaxation. Thus Chinese Bioenergy Qigong was chosen to be the intervention program in this study. Additional details can be obtained from the following webpages:

 
https://www.youtube.com/watch?v=M8b6WkAlLB8;
 
http://info.ck17.org/index.php/chi-kung-demonstration/the-17-motions-of-the-health-sport [37, 38].

Acute is referring to a condition of rapid onset and is of generally brief duration. In this study, the physiological and psychological conditions before and after qigong exercise were evaluated within 30 minutes, so the physiological and psychological effects of Qigong exercise were conceptually defined as acute effects.

### 2.2. Participants

One of the authors in this study, TT Wei, is the senior coach of Chinese Bioenergy Qigong. He announced the information about the study and searched for participants. Optimal qigong practicing environment to conduct the study was taken into consideration. It takes about two months to learn to practice Chinese Bioenergy Qigong. The low frequency of practicing may forget how to practice correctly.

As far back as 1875, in Britain, the Friendly Societies Act enacted the definition of old age as “any age after 50” [39]. The age of 50 years was defined as the working definition of “older” or “old” in World Health Organization (WHO) minimum data set (MDS) project meeting on aging in 2001 in order to take into account the real situation of older persons in developing countries [40]. Furthermore, participants aged 50 to 70 years were regarded as elders in the previous study [41].

From September 23, 2016, to November 5, 2016, elders aged 50 to 90 years from two communities (Tou-Liou and Tan-Mu) in Taiwan without severe medical conditions and practicing Chinese Bioenergy Qigong 5 days a week for at least 3 months were eligible to be recruited in this study in order to make sure the participants can practice qigong correctly and smoothly. Subjects who were unable to read and sign the consent form, had been diagnosed with a major illness (such as acute myocardial infarction, stroke, late-stage cancer, paralysis, and major organ transplantation), were pregnant, or had been diagnosed with a mental disorder (such as anxiety and depression) were excluded.

The study was approved by the Institutional Review Board of Shown Chwan Memorial Hospital, Taiwan (number 1040408). Informed consent was provided by all participants.

### 2.3. Measurements

In order to avoid interoperator variability, the machines were operated by the same skilled operator in this study [42]. The skin electrical conductance was operated by CY Lin, and heart rate variability was operated by CC Wang for all participants. The meridian electrical conductance and heart rate variability data were measured before and after qigong exercise within 30 minutes.

#### 2.3.1. Meridian Electrical Conductance

The participants were rested on a chair for 10 minutes before measurements were recorded. All measurements were obtained on a sunny morning in fall to maintain temperature and moisture consistency. It has been reported that the degree of skin moisture would influence Meridian Energy Analysis Device (MEAD) measurement [25]. To avoid sweat after qigong influence MEAD measurement, all participants were required to dry the body with a dry towel especially wrists and ankle. The electrical conductance of 24 acupuncture points in the wrists and ankles of the 12 left and 12 right meridians was measured using a device (MEAD, 6th generation, Medpex Enterprises, Taiwan). The meridians were as follows: lung (L, H1), pericardium (P, H2), heart (H, H3), small intestine (SI, H4), triple energizer (TE, H5), large intestine (LI, H6), spleen (SP, F1), liver (LIV, F2), kidney (K, F3), urinary bladder (B, F4), gallbladder (G, F5), and stomach (S, F6). The conductivity at the acupuncture point is directly proportional to the amperage of the DC current that flows through the skin when 12 V with an output current of 0–200 uA is applied to the points individually. Higher values indicate higher conductance between the reference electrode, which is 35 mm in diameter and clipped on the left palm, and the acupuncture point, which is measured using a 10 mm diameter cotton with saturated saline solution. The average electrical conductance of 24 meridians in each subject was calculated. The index of sympathetic/vagal balance was defined as the highest average limb electroconductivity value on the dorsal or ventral side, which was divided by the lowest value during MEAD analysis. The yin meridians were lung, pericardium, heart, liver, spleen, and kidney, while yang meridians were small intestine, triple energizer, large intestine, urinary bladder gallbladder, and stomach. The upper meridians indicate those on hands and the lower meridians indicate those on feet. There are 12 meridians each on the left and right sides of the body. The upper to lower body ratio is calculated as the sum of the upper meridians divided by the sum of the lower meridians. The yin to yang ratio is defined as the sum of the yin meridians divided by the sum of the yang meridians. The left to right ratio means the sum of the left meridian divided by the sum of the right meridians [16, 20, 21].

#### 2.3.2. Heart Rate Variability

Five-minute electrocardiography data were recorded immediately after the MEAD measurements. Polar heart-rate monitors (Polar Vantage NV, Polar Electro Oy, Finland) were used to continuously collect heart rate data in the form of RR intervals from all participants before and after one session of qigong exercise. After data collection, HRV parameters were calculated using Kubios HRV (version 2.1; Biosignal Analysis and Medical Imaging Group, University of Eastern Finland, Finland) [27]. The participants' spectral HRV data were expressed in terms of RR intervals, lower frequency (LF), high frequency (HF), normalized LF, and normalized HF.

#### 2.3.3. Questionnaire

Two instruments were adopted in this study. The first was the Short Form 36 (SF-36) health survey and the second was the State-Trait Anxiety Inventory (STAI). Both were self-report questionnaires.

*Short Form 36 Health Survey. *The SF-36 health survey is a self-report survey commonly used as a generic health questionnaire for adults. It covers eight health domains, namely, physical functioning, physical role functioning, bodily pain, general health perceptions, vitality, social role functioning, emotional role functioning, and mental health [30, 43]. The questionnaire includes 36 main questions, which contain several subset questions. These questions are related to the various domains deliberately nested into the instrument to ensure that participants carefully read and assess each question before responding.

*State-Trait Anxiety Inventory. *The STAI contains two subsets, which collect subjective data from participants on their state and trait anxiety in the form of two questionnaires of 20 questions each. These questionnaires are a highly reliable measure of stress and anxiety. State anxiety (STAI-S) is designed to assess individuals' reaction to stress and their emotional state at a particular time, whereas trait anxiety (STAI-T) is related to individuals' personality traits and their stress perceptions. The questions require the participants to rank their current and general feelings toward certain statements among four options of increasing frequency ranging from “almost never” to “almost always.” Higher STAI scores suggest higher anxiety levels [28, 44, 45].

### 2.4. Statistical Analysis

The demographic data were expressed as means and standard deviations. The continuous data before and after one session of qigong exercise were compared using the paired *t*-test. The correlations between the variables were evaluated using the Pearson correlation test. STATA 11 was used for statistical analysis; *p* < 0.05 was considered statistically significant.

## 3. Results

### 3.1. Comparisons of Electrical Conductance of Meridians before and after Qigong Practice

A total of 45 participants (13 men and 32 women; mean age, 65.14 ± 9.38 years) were recruited in this study. All participants were Asian who live in Taiwan. More than half of the participants were married (75.56%). The mean value of education years was 10.62 ± 4.46 years. The mean values of heart rate before and after qigong exercise were 73.24 ± 11.77 and 73.20 ± 11.03 bpm (*p* > 0.05). The electrical conductance of all meridians, except the spleen and bladder meridians (*p* > 0.05), increased significantly (*p* < 0.05) after one session of qigong exercise. The mean values of the electrical conductance of 12 meridians were significantly higher than the baseline values (Table 1). Compared with the baseline values, the upper to lower body ratio and sympathetic/vagal index were significantly improved and close to 1 (*p* = 0.011 and *p* = 0.007, resp.). However, no significant differences were observed in the yin to yang or left to right body balance (*p* = 0.57 and *p* = 0.26, resp.; Table 2).

### 3.2. Comparisons of STAI and SF-36 before and after Qigong Exercise

**Figure 2:**
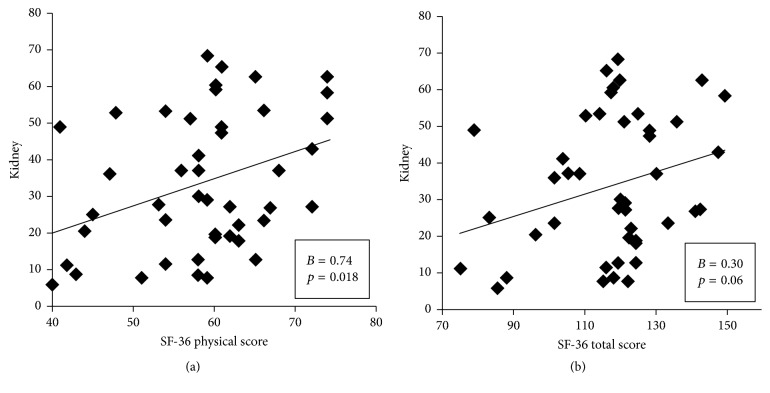
Correlations between kidney meridian electrical conductance and SF-36 physical scores (a) and SF-36 total scores (b) after one session of qigong exercise.

**Figure 3:**
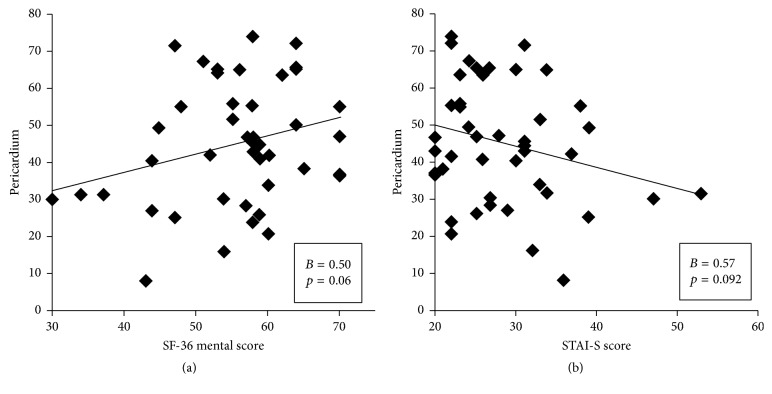
Correlations between pericardium meridian electrical conductance and SF-36 mental scores (a) and STAI-S scores (b) after one session of qigong exercise.

The STAI-S and STAI-T scores and the RR intervals of HRV decreased significantly (*p* < 0.001, *p* = 0.001, and *p* = 0.035, resp.). However, HF, LF, normalized HF, and normalized LF did not differ significantly (*p* = 0.39, *p* = 0.84, *p* = 0.28, and *p* = 0.43, resp.; Table 3). A significant positive correlation was observed between kidney meridian electrical conductance and SF-36 physical scores (*r* = 0.74, *p* = 0.018; Figure 2(a)). Furthermore, nonsignificant positive correlations were observed between kidney meridian electrical conductance and SF-36 total scores (*r* = 0.30, *p* = 0.06; Figure 2(b)). A positive relationship was observed between pericardium meridian electrical conductance and SF-36 mental scores (*r* = 0.50, *p* = 0.06) (Figure 3(a)). By contrast, a negative correlation was observed between pericardium meridian electrical conductance and STAI-S scores (*r* = −0.57, *p* = 0.092; Figure 3(b)).

## 4. Discussion

Qigong is a comprehensive mind-body practice and a low-cost exercise that has been practiced in China for 7000 years. Qigong can be performed anywhere at any time. Studies investigating the sustained benefits of regular qigong practice have revealed that it prevents bone loss, reduces oxidative stress, and increases antioxidant enzymes in middle-aged women and improves quality of life and sleep quality in elderly people [32, 46–48]. Moreover, qigong has been reported to effectively improve balance and strength and promote physical flexibility in healthy adults [49, 50]. TCM describes the meridian system as an essential pathway system and conduit for qi [51]. The core theory of the meridian system plays a crucial role in various therapies, including acupuncture, acupressure, moxibustion, tai chi, and qigong. Major acupuncture points are located on the skin along the 12 main meridian pathways. It is believed that qi is guided and transferred through a connection from the somatic meridian to the internal organs as an integrated system [52–54].

The application of Meridian Energy Analysis Device (MEAD) is of high clinical interest not only in the objective assessment of traditional Chinese medicine but also in predicting the meridian flow of the corresponding organ. However, some technical factors can influence skin electrical impedance, including the size of electrode, the amount of pressure it place on the skin, the accuracy of the acupoint location, environment, and degree of skin moisture [25, 55, 56]. In order to control of the quality of study, the MEAD machine performed automatic digital calibration systems every time before taking measurement to help the operator to exactly control the confounding factors. To avoid interoperator variability, the MEAD machine was operated by the same skilled operator, CY Lin, for all participants. The environment and degree of skin moisture tried to be in the same condition as possible as we can. The MEAD usually indicates constant values in the human body in the absence of external stimulation, visceral abnormalities, of diseases. It has been reported by Nakatani that the reproducibility is 93.2% to support the use of MEAD [24, 25].

The acute physiological and psychological effects of qigong exercise on individual meridians are yet to be reported. After one session of qigong exercise, the electrical conductance of all meridians, except the spleen and bladder meridians, increased significantly in the current study. Our findings are consistent with previous studies, which have revealed that qigong exercise promotes qi and blood circulation to increase physical energy and health [57, 58]. According to TCM, the spleen is responsible for the transformation and transportation of different substances that are the foundation of after-birth existence. Spleen functions are essential in maintaining the digestive power of the body and transforming food into qi and blood [59]. The spleen in TCM is similar to the gastrointestinal system in conventional medicine. Age-related selective decline in gut functions, such as changes in taste, esophageal sphincter motility, gastric emptying, and neurons of gut transit-related mesenteric plexus, may result in malnutrition [60]. Furthermore, aging has been associated with defects in structural and functional mucosal defense, diminished ability to generate protective immunity, and increased incidence of inflammation and oxidative stress; therefore, gastrointestinal disorders occur more frequently in elderly population [60, 61]. In this study, two possible reasons why the spleen meridian electrical conductance did not change significantly are functional deficiencies in the spleen and the long-term effects of age-related gastrointestinal tract states in older adults. In TCM, the bladder meridian is the longest and largest detox pathway. It is often associated with disease symptoms caused by external harmful effects, including cold, wind, fire, dampness, dryness, and summer heat. Because the bladder meridian is considered the most exterior meridian, it is the first to be invaded during external attacks [62]; this means that rapidly modulating its electrical conductance during qigong exercise may be difficult.

We observed a significant positive correlation between kidney meridian electrical conductance and SF-36 physical scores in this study. In TCM, the kidney is considered the foundation for good health and vitality. The kidney is the powerhouse of the body which stores essence (jing) and is the support system for all aspects of organic life. The stored essence comprises congenital and acquired essence and supply reserve energy to any organ. Kidney essence is the material basis for numerous functional activities and is responsible for human growth and development and human vitality [63]. The kidney also absorbs qi [64]. Based on the present findings, we hypothesized that many potential advantages of qigong exercise, such as the enhancement of physical fitness and strength, can be attributed to elevated kidney meridian electrical conductance.

Qigong can affect psychological state, influence the neuroendocrine system, and exert effects on immune cells [9, 65]. This mind-body training has been reported to improve immune functions by stimulating the homeostasis of the sympathetic and parasympathetic nerve systems through hypothalamic action [66–68]. Several studies have investigated the effects of qigong exercise on sleep, distress, anxiety, and depression [29, 69, 70]. In this study, participants were more comfortable, relaxed, and vigorous after one session of qigong exercise. The present findings showed a negative correlation between pericardium meridian electrical conductance and STAI-S scores and a positive correlation between pericardium meridian electrical conductance and SF-36 mental scores. The higher the SF-36 mental scores indicates the healthier the mental status, while the higher the STAI -S scores means the higher the anxiety status. Thus a positive correlation between pericardium meridian electrical conductance and SF-36 mental scores is consistent with a negative correlation between pericardium electrical conductance and STAI -S scores, although nonsignificant. In TCM, the pericardium is the heart's protective sack which is known as a fire-energy organ which protects the heart. Not only does pericardium provide the heart with physical protection, its energy also protects the heart from damage and disruption by excessive emotional energies generated by the other organs including anger from the liver, fear from the kidneys, and grief from the lungs. The pericardium meridian is associated with heart and blood functions. Emotionally, pericardium energy is related to the loving feelings. Because the heart is the seat of spiritual or mental activities, the pericardium meridian is associated with mania and mental illness [71]. Based on the results of the present study, we hypothesized that decreased anxiety and improved mental status due to qigong exercise can be attributed to increased pericardium meridian electrical conductance.

Balanced meridians indicate that an individual is healthy [51, 72]. In our study, one session of qigong exercise improved energy balance between the upper and lower halves of the body. Moreover, the sympathetic/vagal index was significantly improved and closer to 1, indicating an improvement in the autonomic nervous system balance. However, a similar finding was not observed for HRV. Wu et al. reported that skin electrical conductance is a useful tool for detecting subtle noncardiovascular physical responses that may be more sensitive than HRV in analyzing autonomic responses [16]. The present findings are consistent with the results of their study.

The present study had some limitations. First, this is a one-group, pre- and postcomparison study. This study lacked control group of sham intervention to evaluate the placebo effect in this study. Second, the participants were older practitioners; therefore, future studies are suggested to elucidate the effects of qigong exercise across different age groups. Third, blood pressure data were not evaluated and thus the influence of blood pressure on the results was unknown. Fourth, substantial time has to be invested for learning and practicing these mind-body techniques. Fifth, to generalize these findings, further studies using larger sample sizes and/or different kinds of qigong are required. The present findings might be generalized only to older populations who are more health conscious with practicing specific type of qigong exercise, such as Chinese Bioenergy Qigong.

## 5. Conclusions

One session of qigong exercise increased meridian electrical conductance, reduced anxiety, and improved balance in both the autonomic nervous system and the body overall. In conclusion, these findings provide scientific evidence for the acute physiological and psychological effects of qigong exercise in older practitioners. According to the results of this study, Chinese Bioenergy Qigong may be considered as one of the complementary therapies accompanied with conventional medicine to treat the patients with autonomic dysfunction or anxiety. More researches in the future are encouraged to investigate into the possible effects of Chinese Bioenergy Qigong on improving various pathological functions in different population.

## Figures and Tables

**Table 1:** Descriptive statistics of electrical conductance of both sides of 12 meridians before and after qigong practice.

Variables	Before		After		*p *value
	Mean	SD	Mean	SD	
L lung	54.18	19.66	61.10	22.26	0.014^*∗*^
R lung	57.23	21.67	65.84	20.93	0.001^*∗*^
L pericardium	45.79	18.32	51.69	19.14	0.011^*∗*^
R pericardium	44.57	17.45	52.30	17.26	<0.001^*∗*^
L heart	36.87	15.19	44.28	18.33	0.002^*∗*^
R heart	40.09	16.87	46.20	15.97	0.009^*∗*^
L small intestine	50.77	19.37	61.53	20.89	<0.001^*∗*^
R small intestine	50.89	20.04	62.95	21.13	<0.001^*∗*^
L triple warmer channel	60.90	20.89	70.12	19.69	0.001^*∗*^
R triple warmer channel	62.67	21.65	73.29	19.79	<0.001^*∗*^
L large intestine	55.64	20.49	64.90	21.25	0.001^*∗*^
R large intestine	60.05	20.64	69.10	21.34	0.001^*∗*^
L spleen	21.52	12.95	23.44	12.76	0.296
R spleen	20.38	13.71	21.75	13.36	0.552
L liver	32.71	18.60	39.15	17.55	0.004^*∗*^
R liver	30.75	18.09	37.36	18.06	0.002^*∗*^
L kidney	27.14	18.45	35.84	19.52	<0.001^*∗*^
R kidney	22.85	17.03	32.59	18.58	<0.001^*∗*^
L bladder	14.77	11.80	16.80	10.86	0.228
R bladder	14.26	11.20	15.16	10.73	0.559
L gall bladder	21.85	21.85	27.38	15.51	0.001^*∗*^
R gall bladder	20.72	14.23	25.23	15.89	0.009^*∗*^
L stomach	33.70	20.95	39.58	19.23	0.005^*∗*^
R stomach	32.93	19.21	40.78	18.27	0.001^*∗*^
All mean	37.05	14.31	44.98	14.48	<0.001^*∗*^

L: left; R: right. ^*∗*^Significant differences between before and after a qigong exercise.

**Table 2:** Descriptive statistics of electrical conductance balance before and after qigong practice.

Variables	Before		After		*p *value
	Mean	SD	Mean	SD	
Upper/lower balance	2.04	0.94	1.79	0.67	0.011^*∗*^
Index of sympathovagal balance	3.1	1.91	2.48	0.94	0.007^*∗*^
Yin/Yang balance	0.98	0.16	0.97	0.12	0.57
Left/right balance	1	0.1	0.99	0.09	0.26

^*∗*^Significant differences between before and after a qigong exercise.

**Table 3:** Descriptive statistics of STAI, SF-36, and HRV before and after qigong exercise.

Variables	Before		After		*p *value
	Mean	SD	Mean	SD	
STAI-S	32.91	8.40	28.44	7.26	<0.001^*∗*^
STAI-T	36.60	9.21	33.80	9.52	0.001^*∗*^
*SF-36*					
Total score	116.58	15.50	117.18	17.11	0.460
Physical score	58.93	8.16	58.49	8.91	0.430
Mental score	54.76	8.48	55.64	0.15	0.098
*HRV*					
RR interval (ms)	848.22	161.75	809.64	158.02	0.035^*∗*^
HF	847.51	2754.66	1101.3	3256.69	0.39
LF	572.36	1606.11	560.05	1510.56	0.84
HF Ln	4.6	1.78	4.47	1.99	0.28
LF Ln	4.84	1.57	4.77	1.54	0.43

STAI: state-trait anxiety inventory; SF-36: short form 36; HRV: heart rate variability; HF: high frequency; LF: lower frequency; HF Ln: normalized high frequency; LF Ln: normalized lower frequency. ^*∗*^Significant differences between before and after a qigong exercise.

## Abbreviations

STAI: State-Trait Anxiety Inventory
HRV: Heart rate variability
SF-36: Short Form 36
LF: Lower frequency
HF: High frequency
STAI-S: State anxiety
STAI-T: Trait anxiety.

## References

[1] Wang C.-W., Chan C. L. W., Ho R. T. H., Tsang H. W. H., Chan C. H. Y., Ng S.-M. (2013). Effects of Qigong on depressive and anxiety symptoms: a systematic review and meta-analysis of randomized controlled trails. *Evidence-Based Complementary and Alternative Medicine*.

[2] Tsang H. W. H., Cheung L., Lak D. C. C. (2002). Qigong as a psychosocial intervention for depressed elderly with chronic physical illnesses. *International Journal of Geriatric Psychiatry*.

[3] Xiong X., Wang P., Li X., Zhang Y. (2015). Qigong for hypertension: a systematic review. *Medicine*.

[4] Koh T. (1982). Qigong — Chinese Breathing Exercise. *American Journal of Chinese Medicine*.

[5] Sancier K. M. (1999). Therapeutic benefits of qigong exercises in combination with drugs. *The Journal of Alternative and Complementary Medicine*.

[6] Lee M. S., Hong S.-S., Lim H.-J., Kim H.-J., Woo W.-H., Moon S.-R. (2003). Retrospective survey on therapeutic efficacy of Qigong in Korea. *American Journal of Chinese Medicine*.

[7] Chan C. L.-W., Wang C.-W., Ho R. T.-H. (2012). A systematic review of the effectiveness of qigong exercise in Cardiac rehabilitation. *American Journal of Chinese Medicine*.

[8] Chan C. L. W., Wang C.-W., Ho R. T. H. (2012). A systematic review of the effectiveness of qigong exercise in supportive cancer care. *Supportive Care in Cancer*.

[9] Wang C.-W., Ng S.-M., Ho R. T. H., Ziea E. T. C., Wong V. C. W., Chan C. L. W. (2012). The effect of qigong exercise on immunity and infections: a systematic review of controlled trials. *American Journal of Chinese Medicine*.

[10] Shang C. (2001). Emerging Paradigms in Mind–Body Medicine. *The Journal of Alternative and Complementary Medicine*.

[11] Poon C. S., Choy T. T., Koide F. T. (1980). A Reliable Method for Locating Electropermeable Points on the Skin Surface. *American Journal of Chinese Medicine*.

[12] Hwang Y. C. (1992). Anatomy and classification of acupoints. *Probl Vet Med*.

[13] Comunetti A., Laage S., Schiessl N., Kistler A. (1995). Characterisation of human skin conductance at acupuncture points. *Experientia*.

[14] Stux G., Pomeranz B. (1987). Advances in research on the mechanism of acupuncture and moxibustion. *Zhen Ci Yan Jiu*.

[15] Chen J.-X., Ma S.-X. (2005). Effects of nitric oxide and noradrenergic function on skin electric resistance of acupoints and meridians. *The Journal of Alternative and Complementary Medicine*.

[16] Wu S.-D., Gau J.-T., Wang Y.-H. (2011). Ryodoraku as a tool monitoring the effects of walking exercise. *Journal of Chinese Integrative Medicine*.

[17] Colbert A. P., Hammerschlag R., Aickin M., McNames J. (2004). Reliability of the prognos electrodermal device for measurements of electrical skin resistance at acupuncture points. *The Journal of Alternative and Complementary Medicine*.

[18] Huang K.-F., Tang S.-T., Chuang C.-Y., Han W.-R., Lin J.-H., Young S.-T. (2011). Different patterns of dynamic variations on electrical conductances of acupoints between Qi vacuity and Qi non-vacuity after glucose ingestion. *The Journal of Alternative and Complementary Medicine*.

[19] Lee M. S., Jeong S.-Y., Lee Y.-H., Jeong D.-M., Eo Y.-G., Ko S.-B. (2005). Differences in electrical conduction properties between meridians and non-meridians. *American Journal of Chinese Medicine*.

[20] Weng C.-S., Hung Y.-L., Shyu L.-Y., Chang Y.-H. (2004). A study of electrical conductance of meridian in the obese during weight reduction. *American Journal of Chinese Medicine*.

[21] Lee C.-T., Chang Y.-H., Lin W.-Y. (2010). Applications of meridian electrical conductance for renal colic: A prospective study. *The Journal of Alternative and Complementary Medicine*.

[22] Hsu C.-C., Weng C.-S., Liu T.-S., Tsai Y.-S., Chang Y.-H. (2006). Effects of electrical acupuncture on acupoint BL15 evaluated in terms of heart rate variability, pulse rate variability and skin conductance response. *American Journal of Chinese Medicine*.

[23] Roth W. T., Wilhelm F. H., Trabert W. (1998). Autonomic instability during relaxation in panic disorder. *Psychiatry Research*.

[24] Nakatani Y. (1956). Skin electric resistance and Ryodoraku. *J Autonomic Nerve*.

[25] Tsai M.-Y., Chen S.-Y., Lin C.-C. (2017). Theoretical basis, application, reliability, and sample size estimates of a Meridian Energy Analysis Device for Traditional Chinese Medicine Research. *Clinics*.

[26] Lee L. S., Rim Y. H., Jeong D.-M., Kim M. K., Joo M. C., Shin S. H. (2005). Nonlinear analysis of heart rate variability during Qi therapy (external qigong). *American Journal of Chinese Medicine*.

[27] Fong S. S. M., Wong J. Y. H., Chung L. M. Y. (2015). Changes in heart-rate variability of survivors of nasopharyngeal cancer during Tai Chi Qigong practice. *Journal of Physical Therapy Science*.

[28] Wikipedia. State-Trait Anxiety Inventory. https://en.wikipedia.org/wiki/State-Trait_Anxiety_Inventory

[29] Wang C.-W., Chan C. H. Y., Ho R. T. H., Chan J. S. M., Ng S.-M., Chan C. L. W. (2014). Managing stress and anxiety through qigong exercise in healthy adults: a systematic review and meta-analysis of randomized controlled trials. *BMC Complementary and Alternative Medicine*.

[30] Wikipedia. SF-36. https://en.wikipedia.org/wiki/SF-36

[31] Liu W., Schaffer L., Herrs N., Chollet C., Taylor S. (2015). Improved sleep after Qigong exercise in breast cancer survivors: A pilot study. *Asia-Pacific Journal of Oncology Nursing*.

[32] Chow Y. W. Y., Tsang H. W. H. (2007). Biopsychosocial effects of qigong as a mindful exercise for people with anxiety disorders: a speculative review. *The Journal of Alternative and Complementary Medicine*.

[33] Posadzki P., Parekh S., Glass N. (2010). Yoga and qigong in the psychological prevention of mental health disorders: A conceptual synthesis. *Chinese Journal of Integrative Medicine*.

[34] Tsang H. W. H., Fung K. M. T. (2008). A review on neurobiological and psychological mechanisms underlying the anti-depressive effect of qigong exercise. *Journal of Health Psychology*.

[35] Zeng Y., Luo T., Xie H., Huang M., Cheng A. S. K. (2014). Health benefits of qigong or tai chi for cancer patients: a systematic review and meta-analyses. *Complementary Therapies in Medicine*.

[36] Bai Z., Guan Z., Fan Y. (2015). The Effects of Qigong for Adults with Chronic Pain: Systematic Review and Meta-Analysis. *American Journal of Chinese Medicine*.

[37] https://www.youtube.com/watch?v=M8b6WkAlLB8

[38] http://info.ck17.org/index.php/chi-kung-demonstration/the-17-motions-of-the-health-sport

[39] Proposed working definition of an older person in Africa for the MDS Project, http://www.who.int/healthinfo/survey/ageingdefnolder/en/

[40] Kowal P. R., Peachey K., WHO (2001). *Information needs for research, policy and action on ageing and older adults: a report of the follow-up meeting to the 2000 Harare MDS Workshop: indicators for the minimum data set project on ageing: a critical review in sub-Saharan Africa*.

[41] Zheng G., Fang Q., Chen B., Yi H., Lin Q., Chen L. (2015). Qualitative evaluation of baduanjin (Traditional Chinese Qigong) on health promotion among an elderly community population at risk for ischemic stroke. *Evidence-Based Complementary and Alternative Medicine*.

[42] Sharma B., Hankey A., Nagendra H. R., Meenakshy K. B. (2014). Inter-operator variability of electrodermal measure at Jing Well points using AcuGraph 3. *JAMS Journal of Acupuncture and Meridian Studies*.

[43] Ware J. E. (2000). SF-36 health survey update. *The Spine Journal*.

[44] Song C., Ikei H., Igarashi M., Takagaki M., Miyazaki Y. (2015). Physiological and psychological effects of a walk in Urban parks in fall. *International Journal of Environmental Research and Public Health*.

[45] Mavrovouniotis F. H., Argiriadou E. A., Papaioannou C. S. (2010). Greek traditional dances and quality of old people's life. *Journal of Bodywork and Movement Therapies*.

[46] Chen H. H., Yeh M. L., Lee F. Y. (2006). The effects of Baduanjin qigong in the prevention of bone loss for middle-aged women. *American Journal of Chinese Medicine*.

[47] Hsu M.-C., Wang T.-S., Liu Y.-P., Liu C.-F. (2008). Effects of baduanjin exercise on oxidative stress and antioxidant status and improving quality of life among middle-aged women. *American Journal of Chinese Medicine*.

[48] Chen M., Liu H., Huang H., Chiou A. (2012). The effect of a simple traditional exercise programme (Baduanjin exercise) on sleep quality of older adults: a randomized controlled trial. *International Journal of Nursing Studies*.

[49] An B.-C., Wang Y., Jiang X. (2013). Effects of baduanjin exercise on knee osteoarthritis: a one-year study. *Chinese Journal of Integrative Medicine*.

[50] Ruddy K. J., Stan D. L., Bhagra A., Jurisson M., Cheville A. L. (2017). Alternative Exercise Traditions in Cancer Rehabilitation. *Physical Medicine and Rehabilitation Clinics of North America*.

[51] Huang S.-M., Tseng L.-M., Chien L.-Y. (2016). Effects of non-sporting and sporting qigong on frailty and quality of life among breast cancer patients receiving chemotherapy. *European Journal of Oncology Nursing*.

[52] Langevin H. M., Yandow J. A. (2002). Relationship of acupuncture points and meridians to connective tissue planes. *The Anatomical Record*.

[53] Ma S. X., Lee P., Li X. Y., Jiang I., Ma E., Hu J. (2015). Influence of age, gender, and race on nitric oxide release over acupuncture points-meridians. *Scientific Reports*.

[54] Chan S. H. H. (1984). What is being stimulated in acupuncture: Evaluation of the existence of a specific substrate. *Neuroscience & Biobehavioral Reviews*.

[55] Evans W. D., McClagish H., Trudgett C. (1998). Factors affecting the in vivo precision of bioelectrical impedance analysis. *Applied Radiation and Isotopes*.

[56] Ahn A. C., Wu J., Badger G. J., Hammerschlag R., Langevin H. M. (2005). Electrical impedance along connective tissue planes associated with acupuncture meridians. *BMC Complementary and Alternative Medicine*.

[57] Guo H. Z., Zhang S. X., Jing B. S. (1991). The characteristics and theoretical basis of the Qigong maneuver. *Aviation, Space, and Environmental Medicine*.

[58] Chang M.-Y. (2015). The theory and practice of health cultivation qigong exercise in traditional Chinese medicine. *Journal of Nursing*.

[59] Integrated Chinese Medicine Holdings Ltd. Leg Tai Yin Spleen, http://www.shen-nong.com/eng/principles/spleenmeridian.html

[60] Soenen S., Rayner C. K., Jones K. L., Horowitz M. (2016). The ageing gastrointestinal tract. *Current Opinion in Clinical Nutrition & Metabolic Care*.

[61] Rémond D., Shahar D. R., Gille D. (2015). Understanding the gastrointestinal tract of the elderly to develop dietary solutions that prevent malnutrition. *Oncotarget*.

[62] Integrated Chinese Medicine Holdings Ltd. Leg Tai Yang Bladder Meridian. http://www.shen-nong.com/eng/principles/bladdermeridian.html

[63] Integrated Chinese Medicine Holdings Ltd. Kidneys store essence (jing). http://www.shen-nong.com/eng/principles/essencekidneys.html

[64] Integrated Chinese Medicine Holdings Ltd. Kidneys rule the grasping of qi. http://www.shen-nong.com/eng/principles/graspingkidneys.html

[65] Hoffman-Goetz L., Pedersen B. K. (1994). Exercise and the immune system: a model of the stress response?. *Trends in Immunology*.

[66] Vera F. M., Manzaneque J. M., Rodríguez F. M., Bendayan R., Fernández N., Alonso A. (2016). Acute Effects on the Counts of Innate and Adaptive Immune Response Cells After 1 Month of Taoist Qigong Practice. *International Journal of Behavioral Medicine*.

[67] Oh B., Butow P., Mullan B. (2012). A critical review of the effects of medical Qigong on quality of life, immune function, and survival in cancer patients. *Integrative Cancer Therapies*.

[68] Manzaneque J. M., Vera F. M., Maldonado E. F. (2004). Assessment of immunological parameters following a qigong training program. *Med Sci Monit*.

[69] Chan J. S. M., Ho R. T. H., Chung K. F. (2014). Qigong exercise alleviates fatigue, anxiety, and depressive symptoms, improves sleep quality, and shortens sleep latency in persons with chronic fatigue syndrome-like illness. *Evidence-Based Complementary and Alternative Medicine*.

[70] Martínez N., Martorell C., Espinosa L., Marasigan V., Domènech S., Inzitari M. (2015). Impact of Qigong on quality of life, pain and depressive symptoms in older adults admitted to an intermediate care rehabilitation unit: a randomized controlled trial. *Aging Clinical and Experimental Research*.

[71] Integrated Chinese Medicine Holdings Ltd. Arm Jue Yin Pericardium Meridian. http://www.shen-nong.com/eng/principles/pericardiummeridian.html

[72] Nagilla N., Hankey A., Nagendra H. (2013). Effects of yoga practice on acumeridian energies: variance reduction implies benefits for regulation. *International Journal of Yoga*.

